# Supplementary material for: Cognitive Slowing, Dysfunction in Verbal Working Memory, Divided Attention and Response Inhibition in Post COVID-19 Condition in Young Adults
Source: Life (Basel). 2025 May 21;15(5):821. doi: 10.3390/life15050821 (PMC12113594; doi:10.3390/life15050821)

Table S1. Vienna Test System Neuro module (Schuhfried®, Austria): description of cognitive tasks and the measured main and subsidiary variables of cognitive functions

| COGNITIVE FUNCTIONS | TESTED FUNCTIONS | DESCRIPTION | MAIN* AND SUBSIDIARY VARIABLES | Description of the variables |
|---|---|---|---|---|
| PERCEPTIONS AND ATTENTION FUNCTIONS | intrinsic alertness (visual) | Unannounced stimuli (black circle), the respondent must react as quickly as possible to every stimulus. | mean reaction time* | logarithmic mean of the individual reaction times |
| | | | median reaction time | median of all reaction times |
| | | | dispersion of reaction time | exponent of the standard deviation of logarithmic reaction time |
| | | | number of missed reactions | number of stimuli in which no response was made within 1500 ms |
| | | | number of false alarms | number of times a response key was pressed when no stimulus had been presented |
| | divided attention (cross-modal, visual/auditory) | The respondent must react as quickly as possible when the relevant stimulus, a square has to become brighter twice in a row, or a tone becomes softer twice in a row. | mean reaction time* | logarithmic mean of the individual reaction times |
| | | | median reaction time | median of all reaction times |
| | | | number of missed reactions | number of stimuli to which no reaction occurred within 2000 ms |
| | | | number of false alarms | number of times a response key was pressed when no stimulus had been presented |
| | | | number of correct reactions | number of correct reactions to relevant stimuli |
| | | | number of visual missed reactions | number of stimuli in visual channel to which no reaction occurred within 2000 ms |
| | | | number of auditory missed reactions | number of stimuli in auditory channel to which no reaction occurred within 2000 ms |
| | processing speed (Trail Making Test, Part A) | The numbers 1 to 25 are arranged pseudo-randomly on the screen; the task is to tap or click on the numbers in sequential order as quickly as possible. | working time* | time between the correct clicking of two consecutive numbers |
| | | | number of errors* | number is incorrectly clicked while working on the item |

| | | | | |
|---|---|---|---|---|
| **RESPONSE INHIBITION** | go/no-go task | Series of circles and triangles are presented one by one on the screen. Triangles occur frequently; the respondent must respond to them by pressing the green button, which builds up a dominant response tendency. Circles appear rarely and require no response; this means that response inhibition is required. | number of commission errors* | describes how frequently inhibition of no-go stimuli was unsuccessful |
| | | | number of omission errors | reports the number of mitted reactions to go stimuli |
| | | | sensitivity index d′ | overall performance, d′ = z(hits)-z(number of false alarms) |
| | | | mean reaction time | mean reaction time for correct processing go stimuli |
| | | | standard deviation of reaction time | standard deviation of the reaction times for correctly processed go stimuli |
| **EXECUTIVE FUNCTION** | cognitive flexibility (Trail Making Test, Part B) | The numbers 1 to 13 and the letters A to L are arranged pseudo-randomly on the screen; the task is to tap or click alternately and in ascending order as quickly as possible. | working time* | time between the correct clicking of two consecutive numbers or letters |
| | | | difference B-A | difference between the working times in Part B and Part A |
| | | | quotient B/A | quotient of the working times in Part B and Part A |
| | | | number of errors* | number or letter is incorrectly clicked while working on the item |
| | verbal working memory (2-back paradigm) | The test material consists of 100 consonants, which are presented one by one with a presentation time of 1.5 seconds and an interstimulus interval of 1.5 seconds. For each consonant, the respondent must decide whether it is identical to the last-but-one consonant. If it is, the respondent must press the green button on the response panel. If it is not, no button is to be pressed. | correct* | number of correct responses to a target stimulus (total hits) |
| | | | mean time "correct" | mean reaction time for correct responses (mean response time "hits") |
| | | | omitted | number of omitted responses to a target stimulus (total missed stimuli) |
| | | | error/incorrect | number of false positive responses (total false alarms) |
| | | | mean time "incorrect" | mean reaction time for incorrect responses (mean response time "false alarms") |
| | | | working time | working time spent on the test in minutes and seconds |

Table S2. Symptoms of COVID-19 infection in C19 (COVID-recovered without PCC) and PCC (Post COVID-19 Condition) groups (based on the WHO Post COVID Case Report Form)

| | C19 + PCC (n=73) | | | | C19 (n=57) | | | | PCC (n=16) | | | |
|---|---|---|---|---|---|---|---|---|---|---|---|---|
| | Yes, but not present anymore | Yes, still present | Yes, intermittent | No | Yes, but not present anymore | Yes, still present | Yes, intermittent | No | Yes, but not present anymore | Yes, still present | Yes, intermittent | No |
| **fever** | 15/73 | | 1/73 | 57/73 | 12/57 | | 1/57 | 44/57 | 3/16 | | | 13/16 |
| **light fever** | 18/71 | | 3/71 | 50/71 | 16/55 | | 2/55 | 37/55 | 2/16 | | 1/16 | 13/16 |
| **loss of appetite** | 11/73 | 1/73 | 6/73 | 55/73 | 8/57 | 1/57 | 4/57 | 44/57 | 3/16 | | 2/16 | 11/16 |
| **persistent fatigue** | 10/72 | 8/72 | 18/72 | 36/72 | 8/56 | 5/56 | 11/56 | 32/56 | 2/16 | 3/16 | 7/16 | 4/16 |
| **weight loss** | 7/73 | 1/73 | 3/73 | 62/73 | 6/57 | 1/57 | 3/57 | 47/57 | 1/16 | | | 15/16 |
| **can't move and/or feel one side of body or face** | 3/73 | | | 70/73 | 3/57 | | | 54/57 | | | | 16/16 |
| **dizziness/light headedness** | 11/72 | 6/72 | 11/72 | 44/72 | 8/56 | 3/56 | 8/56 | 37/56 | 3/16 | 3/16 | 3/16 | 7/16 |
| **fainting/blackouts** | 3/73 | 1/73 | 3/73 | 66/73 | 2/57 | | | 55/57 | 1/16 | 1/16 | 3/16 | 11/16 |
| **forgetfulness** | 4/73 | 9/73 | 17/73 | 43/73 | 4/57 | 6/57 | 12/57 | 35/57 | | 3/16 | 5/16 | 8/16 |
| **numbness or tingling** | 3/72 | 2/72 | 8/72 | 59/72 | 2/56 | 1/56 | 7/56 | 46/56 | 1/16 | 1/16 | 1/16 | 13/16 |
| **persistent headache** | 6/73 | 3/73 | 10/73 | 54/73 | 4/57 | 2/57 | 5/57 | 46/57 | 2/16 | 1/16 | 5/16 | 8/16 |
| **problems passing urine** | 4/73 | 2/73 | 1/73 | 66/73 | 4/57 | 1/57 | 1/57 | 51/57 | | 1/16 | | 15/16 |
| **problems with balance** | 3/72 | 1/72 | 5/72 | 63/72 | 2/56 | | 3/56 | 51/56 | 1/16 | 1/16 | 2/16 | 12/16 |
| **seizures** | 3/73 | 1/73 | 5/73 | 64/73 | 3/57 | 1/57 | 4/57 | 49/57 | | | 1/16 | 15/16 |
| **slowness of movement** | 4/73 | | 5/73 | 64/73 | 3/57 | | 3/57 | 51/57 | 1/16 | | 2/16 | 13/16 |
| **sleeping less** | 3/73 | 3/73 | 9/73 | 58/73 | 2/57 | 2/57 | 6/57 | 47/57 | 1/16 | 1/16 | 3/16 | 11/16 |
| **sleeping more** | 6/73 | 9/73 | 9/73 | 49/73 | 6/57 | 6/57 | 5/57 | 40/57 | | 3/16 | 4/16 | 9/16 |
| **tremors** | 3/72 | 1/72 | 3/72 | 65/72 | 2/56 | 1/56 | 2/56 | 51/56 | 1/16 | | 1/16 | 14/16 |
| **trouble in concentrating** | 4/73 | 9/73 | 19/73 | 41/73 | 4/57 | 4/57 | 13/57 | 36/57 | | 5/16 | 6/16 | 5/16 |
| **weakness in limbs** | 8/72 | | 3/72 | 61/72 | 6/56 | | 1/56 | 49/56 | 2/16 | | 2/16 | 14/16 |
| **anxiety** | 7/73 | 14/73 | 12/73 | 40/73 | 6/57 | 10/57 | 10/57 | 31/57 | 1/16 | 4/16 | 2/16 | 9/16 |
| **behaviour change** | 8/71 | 2/71 | 10/71 | 51/71 | 7/55 | 1/55 | 6/55 | 41/55 | 1/16 | 1/16 | 4/16 | 10/16 |
| **depressed mood** | 13/73 | 4/73 | 20/73 | 36/73 | 9/57 | 3/57 | 15/57 | 30/57 | 4/16 | 1/16 | 5/16 | 6/16 |
| **loss of interest/pleasure** | 8/73 | 6/73 | 19/73 | 40/73 | 5/57 | 3/57 | 16/57 | 33/57 | 3/16 | 3/16 | 3/16 | 7/16 |

| | | | | | | | | | | | | |
|---|---|---|---|---|---|---|---|---|---|---|---|---|
| **hallucinations** | 2/73 | | 1/73 | 70/73 | 2/57 | | 1/57 | 54/57 | | | | 16/16 |
| **constipation** | 3/73 | 1/73 | 6/73 | 63/73 | 3/57 | | 4/57 | 50/57 | | 1/16 | 2/16 | 13/16 |
| **diarrhoea** | 7/73 | 2/73 | 9/73 | 55/73 | 6/57 | 2/57 | 8/57 | 41/57 | 1/16 | | 1/16 | 14/16 |
| **nausea/vomiting** | 5/73 | | 5/73 | 63/73 | 4/57 | | 4/57 | 49/57 | 1/16 | | 1/16 | 14/16 |
| **problem swallowing** | 2/73 | | 1/73 | 70/73 | 2/57 | | | 55/57 | | | 1/16 | 15/16 |
| **stomach pain** | 2/73 | 1/73 | 7/73 | 63/73 | 2/57 | 1/57 | 4/57 | 50/57 | | | 16 | 13/16 |
| **palpitations** | 4/72 | 3/72 | 8/72 | 57/72 | 3/57 | 2/57 | 7/57 | 45/57 | 1/15 | 1/15 | 1/15 | 12/15 |
| **chest pain** | 9/72 | | 7/72 | 56/72 | 7/57 | | 4/57 | 46/57 | 2/15 | | 3/15 | 10/15 |
| **post-exertional malaise** | 6/71 | | 8/71 | 57/71 | 5/55 | | 3/55 | 47/55 | 1/16 | | 5/16 | 10/16 |
| **problems hearing** | 2/73 | 3/73 | 8/73 | 60/73 | 2/57 | 2/57 | 6/57 | 47/57 | 1/16 | | 2/16 | 13/16 |
| **problems seeing** | 3/73 | 3/73 | 4/73 | 63/73 | 3/57 | 2/57 | 4/57 | 48/57 | | 1/16 | | 15/16 |
| **reduced smell** | 11/72 | 6/72 | 6/72 | 49/72 | 8/57 | 3/57 | 3/57 | 43/57 | 3/15 | 3/15 | 3/15 | 6/15 |
| **reduced taste** | 13/73 | 3/73 | 3/73 | 54/73 | 8/57 | 1/57 | | 48/57 | 5/16 | 2/16 | 3/16 | 6/16 |
| **ringing in ears** | 5/72 | | 9/72 | 58/72 | 3/56 | | 8/56 | 45/56 | 2/16 | | 1/16 | 13/16 |
| **jerking of limbs** | 3/69 | | 6/69 | 60/69 | 2/53 | | 4/53 | 47/53 | 1/16 | | 2/16 | 13/16 |
| **joint pain/swelling** | 9/73 | 2/73 | 6/73 | 56/73 | 6/57 | 1/57 | 3/57 | 47/57 | 3/16 | 1/16 | 3/16 | 9/16 |
| **pain on breathing** | 7/73 | | 4/73 | 62/73 | 4/57 | | 3/57 | 50/57 | 3/16 | | 1/16 | 12/16 |
| **persistent muscle pain** | 5/73 | | 3/73 | 65/73 | 3/57 | | | 54/57 | 2/16 | | 3/16 | 11/16 |
| **problems with gait/falls** | 2/73 | | | 71/73 | 2/57 | | | 55/57 | | | | 16/16 |
| **stiffness of muscles** | 2/73 | | 2/73 | 69/73 | 2/57 | | 1/57 | 54/57 | | | 1/16 | 15/16 |
| **swollen ankles** | 2/73 | | | 71/73 | 2/57 | | | 55/57 | | | | 16/16 |
| **persistent dry cough** | 10/73 | 1/73 | 9/73 | 53/73 | 8/57 | | 7/57 | 42/57 | 2/16 | 1/16 | 2/16 | 11/16 |
| **shortness of breath** | 11/73 | | 8/73 | 54/73 | 6/57 | | 4/57 | 47/57 | 5/16 | | 4/16 | 7/16 |
| **lumpy lesions (on toes/COVID toes)** | 4/73 | | | 69/73 | 3/57 | | | 54/57 | 1/16 | | | 15/16 |
| **skin rash** | | | 5/73 | 68/73 | | | 4/57 | 53/57 | 1/16 | | | 15/16 |
| **dysmenorrhea/erectile dysfunction** | 4/73 | 8/73 | 15/73 | 46/73 | 3/57 | 7/57 | 10/57 | 37/57 | 1/16 | 1/16 | 5/16 | 9/16 |

Table S3. The detailed results of cognitive functions in main and subsidiary variables in the no-C19 (COVID-negative controls), PCC (Post COVID-19 infection) and C19 (COVID-recovered without PCC) groups (Vienna Test System Neuro module; Schuhfried®, Austria).

| **ATTENTION** | **no-C19** (n=34) | **PCC** (n=16) | **C19** (n=57) | $F/\chi^2$ | $p$ | $\eta^2_p/V$ |
|---|---|---|---|---|---|---|
| **Intrinsic alertness** | | | | | | |
| M±SD (PR) | 71.1±27 | 75.2±29.4 | 72.4±25.8 | 0.124 | 0.883 | 0.00 |
| below-average, % | 8.8 | 12.5 | 10.5 | 0.167 | 0.920 | 0.04 |
| *median reaction time* | | | | | | |
| M±SD (raw) | 203.1±24.9 | 199.8±24.9 | 199.6±21.5 | 0.268 | 0.765 | 0.01 |
| M±SD (PR) | 70.5±27.5 | 74.6±27.4 | 72.3±24.9 | 0.135 | 0.874 | 0.00 |
| below-average, % | 11.8 | 6.2 | 3.5 | 2.377 | 0.305 | 0.15 |
| **Divided attention** | | | | | | |
| M±SD (PR) | 36.4±24.4 | 25±23.5 | 31.3±25.5 | 1.201 | 0.305 | 0.02 |
| below-average, % | 41.2 | 62.5 | 57.9 | 3.035 | 0.219 | 0.17 |
| *median reaction time* | | | | | | |
| M±SD (raw) | 437.6±93.8 | 501.6±116.1 | 462.5±108.6 | 2.031 | 0.136 | 0.04 |
| M±SD (PR) | 33.6±23.5 | 22.8±22.6 | 28.5±24.2 | 1.201 | 0.305 | 0.02 |
| below-average, % | 47.1 | 68.8 | 56.1 | 2.119 | 0.347 | 0.14 |
| **Processing speed** | | | | | | |
| M±SD (PR) | 37.2±28.4 | 35.7±32.4 | 36.6±27 | 0.015 | 0.985 | 0.00 |
| below-average, % | 47.1 | 50 | 43.9 | 0.222 | 0.895 | 0.05 |
| *working time* | | | | | | |
| M±SD PR | 37.6±27.4 | 36.3±32 | 36.6±27 | 0.018 | 0.982 | 0.00 |
| below-average, % | 58.8 | 50 | 57.9 | 0.385 | 0.825 | 0.06 |
| **EXECUTIVE FUNCTION** | **no-C19** (n=34) | **PCC** (n=16) | **C19** (n=57) | $F/\chi^2$ | $p$ | $\eta^2_p/V$ |
| **Cognitive flexibility** | | | | | | |
| M±SD (PR) | 54±29.4 | 46.8±27 | 51.6±28.6 | 0.349 | 0.706 | 0.01 |
| below-average, % | 17.6 | 25 | 22.8 | 0.473 | 0.789 | 0.07 |
| *working time* | | | | | | |
| M±SD raw | 23.1±7.2 | 24.8±8.4 | 23.1±5.7 | 0.420 | 0.658 | 0.01 |
| M±SD PR | 53.5±28.6 | 45.6±26.9 | 51±28.3 | 0.428 | 0.653 | 0.01 |
| below-average, % | 17.6 | 25 | 24.6 | 0.657 | 0.720 | 0.08 |
| *difference B-A* | | | | | | |
| M±SD PR | 63.9±31.4 | 57.2±28.5 | 60.5±29.9 | 0.286 | 0.752 | 0.01 |
| below-average, % | 17.6 | 18.8 | 17.5 | 0.657 | 0.720 | 0.08 |
| *quotient B/A* | | | | | | |
| M±SD PR | 64±30.8 | 59.1±29.2 | 64±29.7 | 0.183 | 0.833 | 0.00 |
| below-average, % | 17.6 | 18.8 | 15.8 | 0.657 | 0.720 | 0.08 |
| **Verbal working memory** | | | | | | |
| **M±SD (PR) [correct]** | **47.9±18.6** | **34.4±19.4** | **48.3±17.4** | **3.917** | **0.023** | **0.07** |
| **below-average, %** | **20.6** | **56.2** | **15.8** | **11.637** | **0.003** | **0.33** |
| *mean time "correct"* | | | | | | |
| M±SD raw | 0.67±0.12 | 0.75±0.23 | 0.65±0.13 | 2.656 | 0.075 | 0.05 |
| M±SD PR | 63.3±28.4 | 51.9±36.4 | 66.7±24.4 | 1.783 | 0.173 | 0.01 |
| **below-average, %** | **8.8** | **37.5** | **5.3** | **13.325** | **0.001** | **0.35** |

| | | | | | | |
|---|---|---|---|---|---|---|
| *omitted* | | | | | | |
| M±SD (raw) | 2±2.4 | 3.3.±2.5 | 1.9±2 | 2.779 | 0.067 | 0.05 |
| M±SD PR | 55±26 | 38.9±27.1 | 55.1±24.7 | 2.717 | 0.071 | 0.05 |
| **below-average, %** | **20.6** | **56.2** | **15.8** | **11.637** | **0.003** | **0.33** |
| *error/incorrect* | | | | | | |
| M±SD (raw) | 2.9±3.2 | 3.2±3 | 2.8±3.4 | 0.085 | 0.919 | 0.00 |
| M±SD PR | 55.8±29.1 | 50.9±24.9 | 56.1±26 | 0.245 | 0.783 | 0.01 |
| below-average, % | 17.6 | 12.5 | 12.3 | 0.545 | 0.762 | 0.07 |
| *mean time "incorrect"* | | | | | | |
| M±SD raw | 0.9±0.6 | 0.8±0.4 | 0.7±0.4 | 1.759 | 0.177 | 0.03 |
| **M±SD PR** | **47.6±35.3** | **55.1±34** | **66.2±30.4** | **3.608** | **0.031** | **0.07** |
| below-average, % | 35.3 | 25 | 15.8 | 4.553 | 0.103 | 0.21 |
| *working time* | | | | | | |
| M±SD raw | 4:18:00±0:07:03 | 4:19:56±0:06:25 | 4:17:57±0:07:29 | 0.732 | 0.483 | 0.01 |
| **Response inhibition** | | | | | | |
| M±SD (raw) [commissions] | 5.5±2.8 | 5.6±3.5 | 5.3±3.6 | 0.062 | 0.940 | 0.00 |
| M±SD (PR) | 45.4±23.3 | 45.3±31.5 | 47.7±27.9 | 0.099 | 0.906 | 0.00 |
| below-average, % | 14.7 | 37.5 | 19.3 | 3.580 | 0.167 | 0.18 |
| *number of omissions* | | | | | | |
| M±SD (raw) | 2.3±2.5 | 0.9±1.3 | 2.2±3.1 | 1.649 | 0.197 | 0.03 |
| M±SD (PR) | 30.8±25.5 | 48.3±26 | 33.5±24.1 | 2.878 | 0.061 | 0.05 |
| below-average, % | 50 | 25 | 38.6 | 2.957 | 0.228 | 0.17 |
| *sensitivity index d'* | | | | | | |
| M±SD (raw) | 2.9±0.7 | 3.3±0.8 | 3±1 | 0.637 | 0.531 | 0.01 |
| M±SD (PR) | 38.3±25 | 46.6±28.8 | 43±27.7 | 0.599 | 0.551 | 0.01 |
| below-average, % | 32.4 | 25 | 24.6 | 0.696 | 0.706 | 0.08 |
| *mean reaction time* | | | | | | |
| M±SD (raw) | 0.285±0.045 | 0.292±0.044 | 0.277±0.035 | 1.066 | 0.348 | 0.02 |
| M±SD (PR) | 35.6±25.1 | 30.3±22.7 | 40±23.9 | 1.116 | 0.331 | 0.02 |
| below-average, % | 41.2 | 56.2 | 35.1 | 2.348 | 0.309 | 0.15 |
| *standard deviation of reaction time* | | | | | | |
| **M±SD (raw)** | **0.07±0.03** | **0.09±0.03** | **0.07±0.03** | **3.461** | **0.035** | **0.06** |
| **M±SD (PR)** | **42.3±25.2** | **31.6±29.2** | **53.7±27.9** | **4.763** | **0.010** | **0.08** |
| **below-average, %** | **29.4** | **56.2** | **15.8** | **10.857** | **0.004** | **0.32** |

*Notes.* Statistically significant results ($p < 0.05$) are in bold.

Figure S1. Study sample and group selection

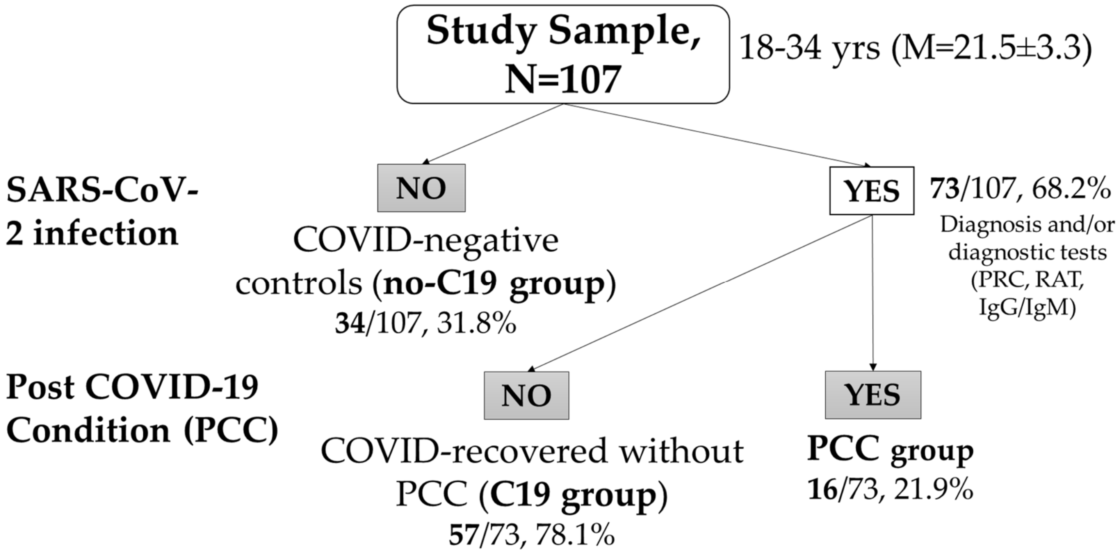

Supplement: Supplementary file 1 [file life-15-00821-s001.zip › life-3653189-supplementary.pdf]
